# The axis of complement C1 and nucleolus in antinuclear autoimmunity

**DOI:** 10.3389/fimmu.2023.1196544

**Published:** 2023-06-09

**Authors:** Shan Wu, Junjie Chen, Boon Heng Dennis Teo, Seng Yin Kelly Wee, Ming Hui Millie Wong, Jianzhou Cui, Jinmiao Chen, Khai Pang Leong, Jinhua Lu

**Affiliations:** ^1^ Department of Microbiology and Immunology, Yong Loo Lin School of Medicine, National University of Singapore, Singapore, Singapore; ^2^ Immunology Translational Research Program, Yong Loo Lin School of Medicine, National University of Singapore, Singapore, Singapore; ^3^ Singapore Immunology Network, Agency for Science, Technology and Research, Singapore, Singapore; ^4^ Department of Rheumatology, Allergy and Immunology, Tan Tock Seng Hospital, Singapore, Singapore

**Keywords:** ANA, SLE, nucleolin, GAR/RGG, alarmin, nucleolus autoimmunity, complement C1

## Abstract

Antinuclear autoantibodies (ANA) are heterogeneous self-reactive antibodies that target the chromatin network, the speckled, the nucleoli, and other nuclear regions. The immunological aberration for ANA production remains partially understood, but ANA are known to be pathogenic, especially, in systemic lupus erythematosus (SLE). Most SLE patients exhibit a highly polygenic disease involving multiple organs, but in rare complement C1q, C1r, or C1s deficiencies, the disease can become largely monogenic. Increasing evidence point to intrinsic autoimmunogenicity of the nuclei. Necrotic cells release fragmented chromatins as nucleosomes and the alarmin HMGB1 is associated with the nucleosomes to activate TLRs and confer anti-chromatin autoimmunogenecity. In speckled regions, the major ANA targets Sm/RNP and SSA/Ro contain snRNAs that confer autoimmunogenecity to Sm/RNP and SSA/Ro antigens. Recently, three GAR/RGG-containing alarmins have been identified in the nucleolus that helps explain its high autoimmunogenicity. Interestingly, C1q binds to the nucleoli exposed by necrotic cells to cause protease C1r and C1s activation. C1s cleaves HMGB1 to inactive its alarmin activity. C1 proteases also degrade many nucleolar autoantigens including nucleolin, a major GAR/RGG-containing autoantigen and alarmin. It appears that the different nuclear regions are intrinsically autoimmunogenic by containing autoantigens and alarmins. However, the extracellular complement C1 complex function to dampen nuclear autoimmunogenecity by degrading these nuclear proteins.

## 1 Introduction

Our knowledge of autoimmune diseases mostly began with the discovery of the lupus erythematosus (L.E.) cell phenomenon (1). Historically, lupus was considered a skin disease (2). At the juncture of the 19th and 20th centuries, it was found to affect visceral organs with female preponderance (3, 4). L.E. cells are phagocytes in SLE patient bone marrows that contain, besides their endogenous nuclei, additional nuclear fragments (1). Research has found that in the presence of SLE patient sera, L.E. cells could form between normal phagocytes and nuclei (5, 6), and the serum L.E. factors were antinuclear autoantibodies (ANA) of heterogeneous specificities (**Table 1**) (8, 46). The 1971 and 1982 SLE diagnosis criteria included L.E. cells that are replaced in the 2012 and 2019 criteria by specific ANA (47–50).

**Table 1:** Major IIF patterns stained with ANA.

IIF patterns (7)		Autoantigens (Ref)	Function
Homogeneous		dsDNA, nucleosome, histones (8–10)	Chromatin network
Nuclear speckled	Coarse speckled	hnRNP, U1RNP, Sm, RNA polymerase III (8, 11–14)	Splicesomes
	Fine speckled	SS-A/Ro, SS-B/La (8, 15)	Non-mitotic cells
Centromere		CENP-A, CENP-B (16, 17)	Chromatin
Nucleolar	Homogeneous	PM/Scl-75, PM/Scl-100, Th/To, NPM-1, NCL, No55/SC65 (18–24)	GC
	Clumpy	U3-snoRNP, fibrillarin (8, 21, 25)	DFC
	Punctate	RNA Pol I, UBF (21, 26, 27)	FC
Nuclear envelope	Smooth	Lamins, lamin-associated proteins (28–31)	Nuclear lamina
	Punctate	nuclear pore complex (32, 33)	NPC
Nuclear dense fine speckled		DFS70/LEDGF (34–36)	n.a.
Multiple nuclear dots		PML proteins (37, 38)	PML body
Few nuclear spots		Coilin, SMN (39–41)	Coiled body, Cajal body
PCNA-like		PCNA (42, 43)	DNA replication
CENP-F-like		CENP-F (44, 45)	kinetochore

Refer to the ICAP International Consensus on ANA patterns for characteristic IIF images.

ANA are commonly measured by indirect immunofluorescence (IIF) microscopy, giving an overall ANA titer and a fluorescent pattern (8, 51). In the current 2019 criteria, a minimal ANA titer of 1/80 is adopted as the entry criterion (50). Single ANA specificities are also adopted in the diagnosis of SLE and other systemic autoimmunity, e.g., anti-Smith (Sm) antigen (SLE), anti-Ro/SSA and anti-La/SSB (Sjogren’s syndrome or SjS), anti-U1-ribonucleoprotein (U1-snRNP) for mixed connective tissue disease (MCTD), and anti-topoisomerase I (Slc70) for systemic sclerosis (SSc) (8, 46, 52). Early evidence that ANA are pathogenic was the observation that antibodies for double-stranded DNA (anti-dsDNA) appeared in the blood before SLE disease flare and then precipitated out of blood circulation when dsDNA surged and active disease developed (53).

Patient sera can give heterogenous IIF patterns, e.g., homogeneous, speckled, nucleolar, centromere, or others (**Table 1**) (8, 51). These patterns are systemically named following an international consensus (7). Based on this classification scheme, each pattern is supported by one or more specific ANA-reactive antigens (9–13, 15–20, 25, 26, 28–40, 42–45). The homogeneous pattern is the most common, followed by the speckled and nucleolar patterns (51). Within the nucleolus, ANA can stain homogeneous, clumpy, or punctate patterns. As detailed later, these nucleolar IIF patterns correspond to three distinct nucleolar regions, i.e., the granular component (GC, homogeneous), the dense fibrillar component (DFC, clumpy), and the fibrillar center (FC, punctate) (54–56). Each nucleolar IIF pattern reflects specific antigens targeted by the patient ANA. While certain antigen-specific ANA show disease specificity such as anti-Sm antigen for SLE, IIF patterns are generally shared among different diseases (57). The prevalence of each IIF pattern can vary depending on the study populations, but the homogeneous and speckled patterns consistently dominate these patterns followed by the nucleolar pattern (e.g., 51, 57, 58).

## 2 The functions of the nucleoli

**Figure 1:**
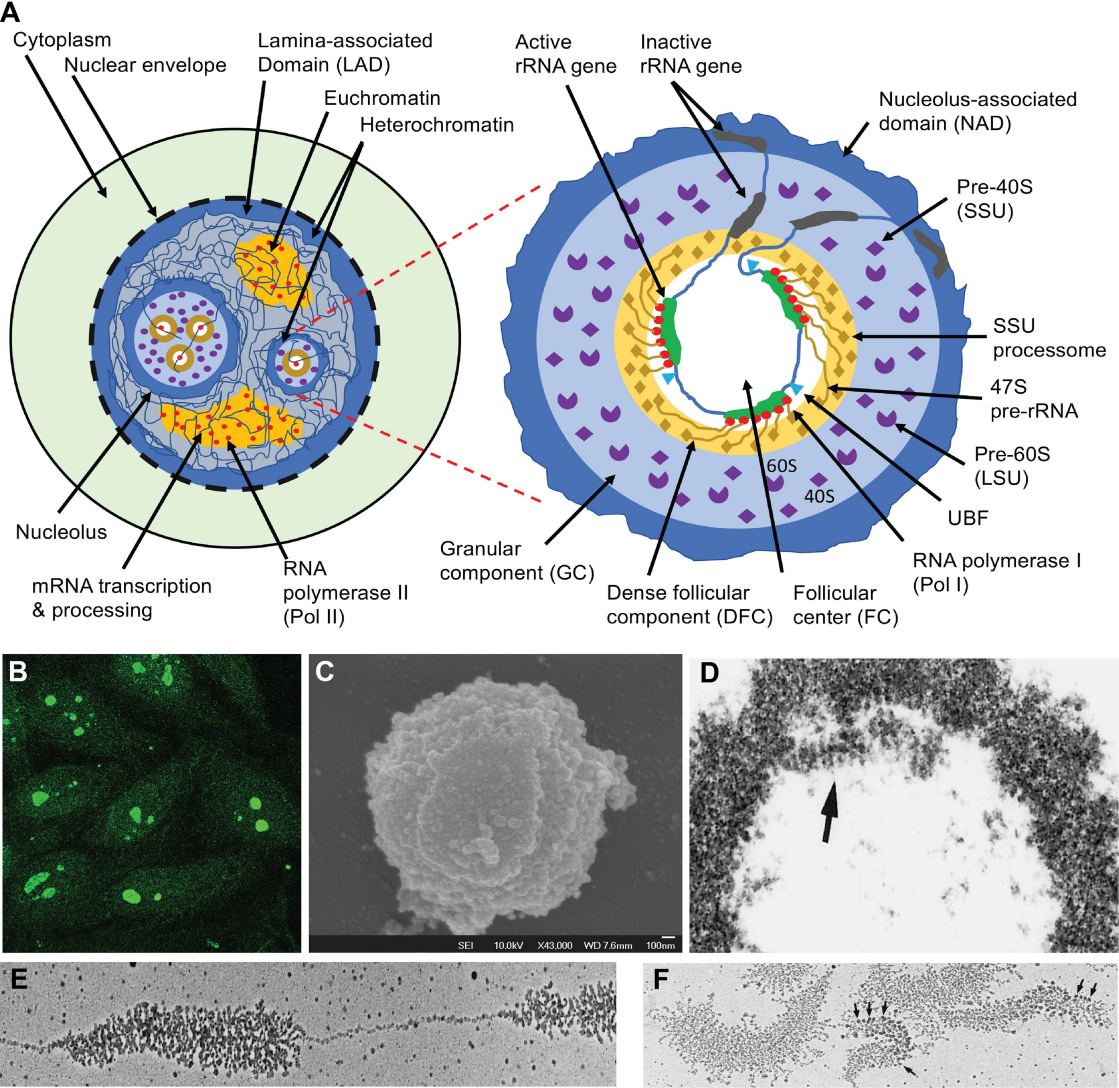
The nucleus, nucleolus, and ribosome biogenesis. **(A)** Schematic illustration of functional nuclear regions. The left panel highlights the nucleus which is partitioned from the cytoplasm with the nuclear envelope which rests in a dense layer of peripheral nuclear heterochromatin, which is also known as lamina-associated domain (LAD). LAD functions as a major nuclear chromatin scaffold. The nucleoli are distinct nuclear regions that are surrounded by a layer of dense heterochromatin. Between this heterochromatin are loose euchromatin regions where mRNA is transcribed by Pol II and processed by complex machinery. The right panel highlights the structure of a nucleolus. The dense layer of heterochromatin that cover each nucleolus is also known as the nucleolus-associated domain (NAD). Inside the enclosed nucleolar region, there are three distinct regions. The rRNA genes and the transcription machinery (Pol I, UBF, etc.) are localized in the follicular center (FC). The rRNA genes are transcribed; the transcripts (47S pre-rRNA) and their processing machinery form the dense follicular component (DFC), and the finished transcripts complete most of their assembly with 79-80 ribosomal proteins (r-proteins) into the 40S small subunit (SSU) and 60S large subunit (LSU) of ribosomes in the large granular component (GC). **(B)** Some SLE patients develop ANA which predominantly reacts with the nucleoli (7). **(C)** Nucleoli can be isolated from the nucleus through sonication, which breaks the chromatin connections between the nucleolar surface heterochromatin layer and the rest of the chromatin network. The image is a nucleolus viewed by scanning electron microscopy (59). **(D)** Electron micrograph of nucleolus isolated from the *locusta* oocytes with the arrow pointing to the DFC region. **(E)** Electron micrograph of a spread *locusta* oocyte nucleolus to show the tandem rRNA genes and the ~ 100 pre-rRNA transcripts that stem from each rRNA gene. **(F)** Electron micrographs of multiple rRNA genes and their transcripts. Arrows point to the rRNA processing machinery corresponding to the SSU processomes. Panels **(D-F)** are reproduced with permission from Scheer et al. (60).

A nucleolus is formed organically around one or more actively transcribed rRNA genes (56). This includes pre-rRNA transcription by RNA polymerase I (Pol I), its processing by U3-snoRNPs and other snoRNPs, and mature rRNA assembly with 79-80 ribosomal proteins into the 40S and 60S pre-ribosomes (**Figure 1**) (61, 62). Ribosomes are then transported into the cytoplasm for protein translation (63). An animal cell nucleus usually contains 1-3 nucleoli but faster-growing cells, e.g., cancer cells, have larger and more numerous nucleoli, making rRNA transcription a target in cancer therapy (64).

The rRNA genes exist variably in many copies in each eukaryotic cell (56, 65). In human cells, the number of rRNA genes can also vary substantially among individuals (315 ± 104) (66, 67), being tandemly clustered head-to-tail on the short arms of the five acrocentric chromosomes (i.e., chromosomes 13, 14, 15, 21, and 22) (65). During mitosis, rRNA transcription ceases, and the majority of the rRNA processing machinery disperses, leaving only residual transcription machinery on the rRNA genes to form the ‘seed’ nucleolar organizer regions (NOR) (56, 65, 68). Some dispersed nucleolar proteins relocate to the surface cortexes of mitotic chromosomes (69, 70). When cells exit mitosis and the rRNA genes resume transcription, NORs expand *de novo* into active nucleoli (68). In these interphase cells, *in situ* NOR-like structures can be induced by inhibiting rRNA transcription (68, 71, 72).

The nucleolus also functions as an inner nuclear scaffold for the chromatin network. The peripheral nuclear scaffold is provided by the nuclear lamina (73), which assembles a dense layer of nuclear surface heterochromatin known as the lamina-associated domain (LAD) (74). Each nucleolus is also surrounded by a dense layer of heterochromatin known as nucleolus-associated domain (NAD) (75, 76). These are transcriptionally inactive chromatin regions that are important in chromatin organization or compartmentalization (77, 78).

Thirdly, the nucleolus may also exhibit multiple other functions (79). Many molecules transit through the nucleolus during cellular stress, e.g., viral infection (80), metabolic disruption (81), and UV stimulation (82). Nucleolus-related functions are unknown for most of these proteins (83).

## 3 The structure of the nucleolus

Nucleoli are dense and visible under light microscopes. By transmission electron microscopy, three distinct regions are found in each nucleolus: one or more FC regions each surrounded by a dense layer of DFC (54, 55), and these are embedded in a greater GC region that borders the outer nucleoplasm through a heterochromatin rim (**Figure 1A**) (56). The FC region contains the rRNA genes and the RNA polymerase I (Pol I) transcription machinery, including a key transcription factor, the upstream binding factor (UBF) (84–87). An active rRNA gene is simultaneously transcribed by approximately 100 Pol I and, therefore, many pre-rRNA transcripts of varying lengths stem from each active rRNA gene, like tree brunches (**Figures 1D–F**) (60, 88, 89). At the 5’ end of each pre-rRNA transcript, a complex machinery is attached that processes the transcript into mature 28S, 18S, and 5.8S rRNAs (**Figures 1A, F**) (54, 90, 91). These pre-rRNAs and their processing machinery form the DFC region. In the GC region, the processed rRNAs assemble with ribosome proteins (r-proteins) to form the 40S and 60S ribosome subunits, and these are transported to the cytoplasm (61, 62).

### 3.1 The FC region

In this nucleolar region, the rRNA genes are constitutively associated with UBF and the Pol I transcription machinery which, in quiescence, form NORs but they expand into nucleoli during active rRNA transcription (56). The 43-Kb human rRNA gene is first transcribed into a 47S pre-rRNA (92), which is then processed in DFC into mature 18S, 5.8S, and 28S rRNA for assembly with r-proteins (56, 93–95). UBF is a master organizer for these tandem rRNA genes. It binds to an upstream control element (UCE) situated at -156 to -107 bp of each rRNA gene promoter region to initiate the Pol I holoenzyme formation (84). Besides, UBF also binds broadly to other regions in the rRNA gene and organizes rRNA gene configuration with a histone-like function (86, 87).

### 3.2 The DFC region

**Figure 2:**
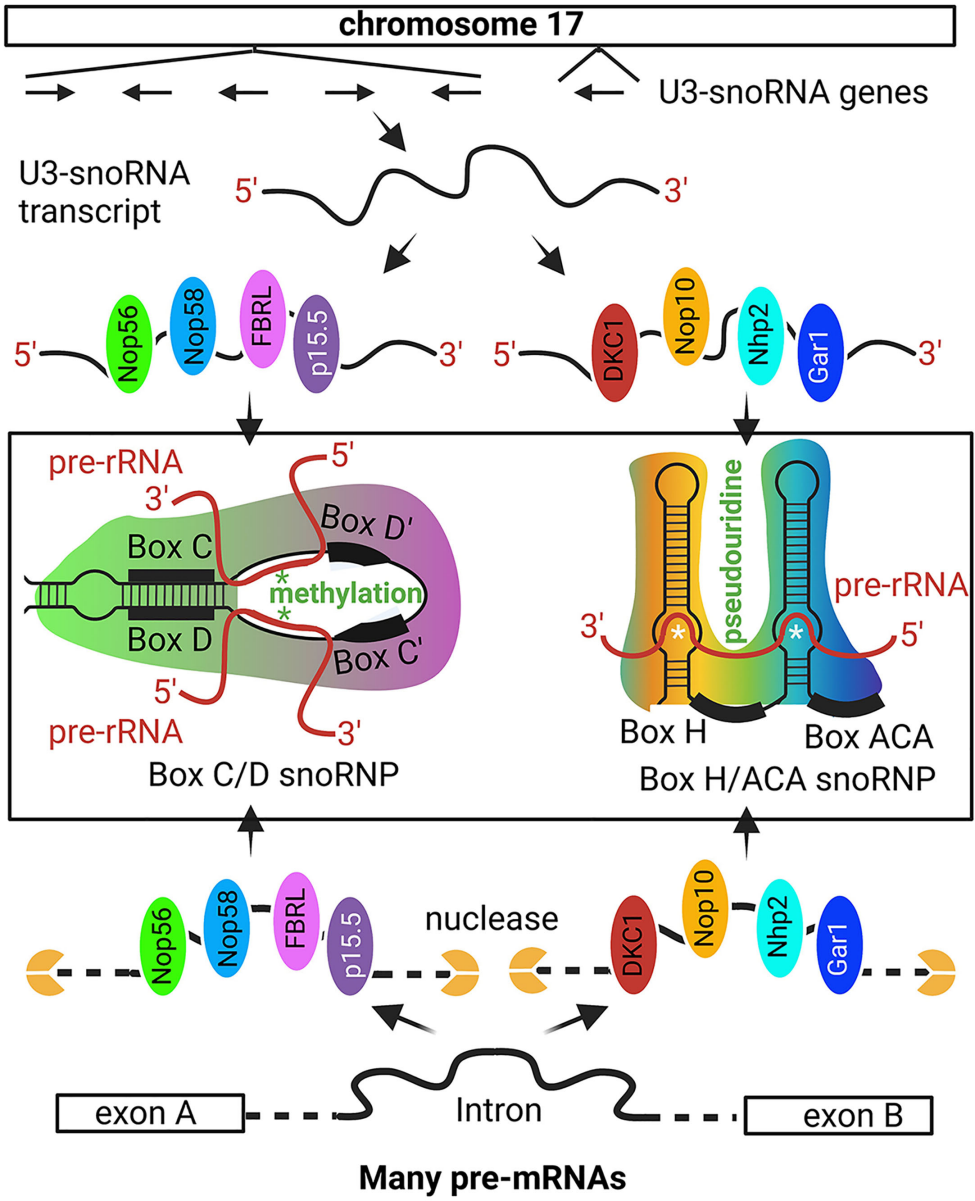
Non-intronic human U3-snoRNA genes on chromosome 17. The majority of snoRNAs are derived from pre-mRNA introns but a small number of U3-snoRNA genes with Pol II promoters are present on chromosome 17. The six snoRNA transcribed from chromosome 17 are bound by distinct protein sets, i.e., the p15.5, Nop56, Nop58, and FBRL set or the Nhp2, Nop10, Gar1, and DKC1 set, to form the C/D and H/ACA box U3 snoRNP, respectively. The C/D box U3 snoRNA binds to specific sequences on pre-rRNA for FBRL to methylate rRNA at specific nucleotides. The H/ACA U3 snoRNA binds to selected pre-rRNA sequences for DKC1 to convert specific uridine into pseudouridine. Besides the six snoRNA genes on chromosome 17, most other snoRNAs are derived from intron sequences spliced out from pr-mRNA. Some intron sequences have the specific features to recruit the p15.5, Nop56, Nop58, and FBRL protein set or the Nhp2, Nop10, Gar1, and DKC1 protein set, which protect these intron regions from nuclease degradation and ultimately form the snoRNPs [Ref (96, 97)]. '*' site of methylation or pseudouridine generation.

The 47S pre-rRNA is simultaneously processed during transcription which includes methylation, pseudouridylation, and cleavage (56, 61, 62, 95). When nucleoli were isolated from the oocytes, they spread like tandem ‘Christmas trees’ along the rRNA gene ‘stem’ under the electron microscope (60, 88, 89). The 5’ processing machinery, including the ribosome small subunit (SSU) processome, were viewed as terminal balls (89). U3-snoRNPs are key SSU elements that methylate and pseudouridylate specific bases in rRNA (**Figure 2**) (96, 98, 99).

U3 snoRNPs include two distinct groups, i.e., box C/D and box H/ACA snoRNPs (96, 100, 101). A box C/D snoRNP contains a guide C/D snoRNA and four core proteins, i.e., SNU13 (NHP2L1), NOP56, NOP58, and fibrillarin (FBRL), and a H/ACA box snoRNP contains a H/ACA box snoRNA and four different core proteins, i.e., NOP10, GAR1, NHP2, and DKC1 (**Figure 2**) (96, 97). In the C/D box snoRNP, FBRL is a ribose 2’-O-methyltransferase, and in H/ACA snoRNPs, DKC1 is a pseudouridine synthase. The guide RNAs target the SnoRNPs to specific pre-rRNA sites so that specific nucleotides are methylated or specific uridine is converted into pseudouridine (96, 100, 102, 103). FBRL is an autoantigen in SSc patients (104).

Most snoRNAs originate from the introns of pre-mRNAs (97, 105–107), with few being transcribed from their own promoters (**Figure 2**) (108). snoRNAs are 60-170 bp RNA fragments and more than 1,000 have been predicted in the human genome (100–102). During pre-mRNA splicing, some introns are protected by snoRNA core proteins from exonuclease degradation, and these are further processed into mature snoRNPs (109, 110). Each C/D box or H/ACA box snoRNA is protected by four core proteins (**Figure 2**) (96, 107, 111).

### 3.3 The GC region

While the FC region is defined by the tandem rRNA genes (56, 84) and the DFC region is defined by the 47S pre-rRNA (88, 89, 112), a defining scaffold for the GC region is not apparent. NCL and NPM1 are highly abundant in the GC region which could be part of the scaffold (113, 114). The processed rRNAs assemble with r-proteins in the GC region (56, 75, 76). NCL facilitates SSU docking on pre-rRNA (115). NPM1 is a molecular chaperone of the nucleolus (116). Both NCL and NPM1 are autoantigens.

## 4 ANA, DNA, and RNA

The homogeneous IIF pattern is largely attributed to ANA binding to the chromatin network, e.g., dsDNA or histones (8). The speckled pattern corresponds to sites of mRNA transcription and processing (117, 118). The nucleolus accommodates ribosome biogenesis (56, 63). For these nuclear regions to elicit self-reactive antibodies, they inevitably involve aberrant innate and adaptive immune responses that lead to B cell production of class-switched IgG class ANA (119). In a healthy individual, 5-20% of peripheral naïve B cells are likely to be self-reactive or polyreactive (120, 121). These B cells can become pathogenic ANA-producing B cells in SLE patients (122). This requires nucleus-reactive CD4 T cell help for which adjuvant signals are necessary.

### 4.1 DNA

In the nucleus, DNA is primarily embedded in the chromatins configurated by histones and additional non-histone DNA-binding proteins. High mobility group box 1 (HMGB1) is a major non-histone DNA-binding protein, and it is also an alarmin that activates innate immunity through Toll-like receptors (TLRs) (123, 124). HMGB1 can be secreted by live cells or passively released by necrotic cells (123). When it is released from secondary necrotic cells in association with fragmented chromatins (nucleosomes), it confers immunogenicity to these known nuclear autoantigens (125).

### 4.2 mRNA

While the nucleolar DFC regions are formed from pr-rRNA and its processomes, the speckled regions are formed from pre-mRNA and its processing machinery, e.g., the two ribonucleoprotein complexes Smith antigen (Sm) and SSA/Ro. Purified Sm antigen can induce self-reactive autoantibodies in mice without additional adjuvant (126). This is because its U1-snRNA element is an endogenous adjuvant that activates TLR7 (127). U1-snRNA itself is also an autoantigen (126). U1-snRNP can activate the NOD-like receptor family, pyrin domain-containing 3 (NLRP3) inflammasomes (128). The snRNA components in SSA/Ro60 also activate TLR7 (129). These snRNA alarmins could confer sufficient autoimmunogenecity to these RNPs to cause B and T cell activation.

### 4.3 rRNA

In the nucleolus, ANA target mostly snoRNP components (21, 59, 130), but snoRNA has not been reported as autoantigens or adjuvants (127, 128). U3-snoRNPs are dominant snoRNPs in the nucleolus (96, 100, 101). Recently, two of the U3-snoRNP protein components have been found to contain alarmin or adjuvant activities, i.e., FBRL and GAR1 (**Figure 2**) (131). Their alarmin activities are conferred by their GAR/RGG motifs which were first discovered in NCL to activate TLR2 and TLR4 (131). The nucleolus contains the most numerous nuclear autoantigens, and nucleolus-reactive naïve B cells are also prevalent in healthy individuals (120, 121). Whether NCL, FBRL, and GAR1 confer sufficient immunogenicity to nucleolar antigens to induce self-reactive antibodies remains to be determined (131). Some transit extranucleolar molecules could also confer nucleolus autoimmunogenecity (83). For example, the EBV virus appears to confer autoimmunogenicity to the speckled region through cross-reactivity with SSA/Ro, Sm, and DNA (132–135).

## 5 Antinucleolar autoantibodies (ANoA)

ANoA are frequently found in SSc or scleroderma patients (21, 130, 136, 137). However, they are not sufficiently specific for SSc diagnosis (14). For example, ANoA for Th/To and U3-snoRNP are also developed in other autoimmune diseases (138, 139). ANoA are also prevalent in SLE and SjS patients (48, 49, 140). ANoA frequently target ribonucleoprotein (RNP) or protein complexes (130). NCL, NPM1, and UBF are exceptions (21, 130).

NCL is not a well-studied autoantigen, but its autoantigenicity was shown on a 25-autoantigen array study in which NCL was the 4th most prominent SLE patient autoantigen following dsDNA, ssDNA, and Ro-52/SSA (22). In TLR7^hi^ SLE patients, NCL was the most prominent protein autoantigen after dsDNA and ssDNA (22). NZBxW F1 and MRL/lpr mice spontaneously develop SLE following aging. In these mice, NCL-reactive antibody was detected early before other common autoantibodies (141), implying that NCL could induce its self-reactive antibody. This view is supported by its intramolecular GAR/RGG alarmin motif (131). Likewise, FBRL could also induce its self-reactive antibodies.

NPM1-reactive ANA develop in SSc (23), SLE (142), and various other systemic autoimmune diseases (143, 144). UBF is mostly targeted by autoantibodies in SSc patients (27, 145, 146). Some cancer patients develop ANA that most consistently target NPM1 and UBF (147–149). Coilin is the master organizer of Coiled bodies and is also a well-known autoantigen (41, 150, 151). snRNPs and snoRNPs mature in these small nuclear bodies before being released to the nucleoplasm and nucleoli, respectively (150). Coiled bodies are often conjunct to nucleoli and therefore coilin is also a nucleolar autoantigen (152).

Nucleolar exosomes cleave pre-rRNA during assembly with r-proteins and most members of these protein complexes are autoantigens (24, 153, 154). RNase P and MRP are abundant autoantigenic RNPs in the nucleolus (155, 156). Approximately 60% of nucleolar autoantigens are snoRNPs and the remaining 40% are proteins like NCL, NPM1, and UBF (139). Some nucleolar U8 and U22 snoRNPs are autoantigenic (139). rRNA is also targeted by autoantibodies in MRL/lpr mice and some SLE patients (157, 158). The abundance of nucleolar autoantigens and endogenous alarmins make these nuclear regions potential initiators in ANA production.

## 6 Genetic predisposition in ANA induction

**Figure 3:**
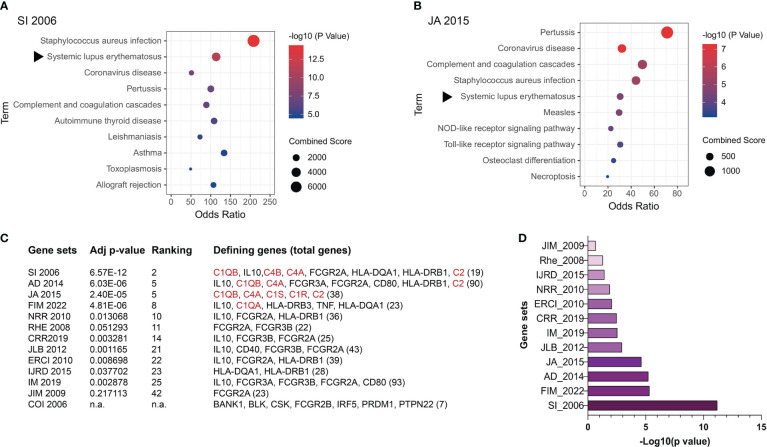
Genetic contributions to SLE pathogenesis. SLE risk genes have been identified based on evidence obtained through genome-wide association studies (GWAS), case reports, and other methods. Here SLE risk gene sets were extracted from 13 articles and their relevance to SLE was assessed through gene ontogeny (GO) analysis. **(A)** GO analysis of the SI 2006’ SLE risk gene set (165). **(B)** GO analysis of the `JA 2015’ SLE risk gene set (169). The rest of the gene sets included in this study are AD 2014 (170), FIM 2022 (171), NRR 2010 (172), RHE 2008 (167), CRR 2019 (173), JLB 2012 (174), ERCI 2010 (175), IJRD 2015 (166), IM 2019 (168), JIM 2009 (176), and COI 2006 (177). **(C)** Relevance of SLE as a disease to the 13 SLE risk gene sets analyzed. Confidence in the level of SLE relevance is indicated by the adjusted p values. The subgroup of SLE risk genes that were identified to derive the p values and SLE ranking positions among other relevant pathways are listed with the total number of SLE risk genes in each set being included at the end of the gene list in the bracket. Complement genes are highlighted in red. n.a. SLE was not identified as one pathway in the COI 2006 gene set. **(D)** Adjusted p values of the selected SLE risk gene sets except for the COI 2006 gene set.

ANA are a hallmark of SLE (50), making this disease a suitable model for dissecting the molecular causes of these autoantibodies. However, most SLE patients present a polygenic disease for which more than 50 risk genes or non-coding loci have been identified albeit they mostly represent weak SLE risks with low SLE specificity (159–168). These risk genes mostly represent immunological pathways broadly underlying infectious and inflammatory diseases (**Figure 3**). We selected 13 published SLE risk gene sets for gene ontogeny (GO) analysis (165–177), and found that in the most SLE-specific gene sets, SLE was only ranked the 2nd most significant pathway following the *Staphycoccous aureus* infection pathway (**Figure 3A**). The next highest ranking for SLE (5th) was found in the `JA 2015’ gene set (**Figure 3B**). SLE was ranked outside the top 10 pathways in seven gene sets (**Figures 3C, D**). With the `COI 2006’ gene set, SLE was not identified as a relevant pathway (**Figure 3C**).

An important observation was that the four gene sets in which SLE was ranked higher than the 10th position all contained one or more complement proteins, i.e., C1q, C1r, C1s, C4, and C2 (178, 179). The remaining nine gene sets all lacked complement genes.

**Figure 4:**
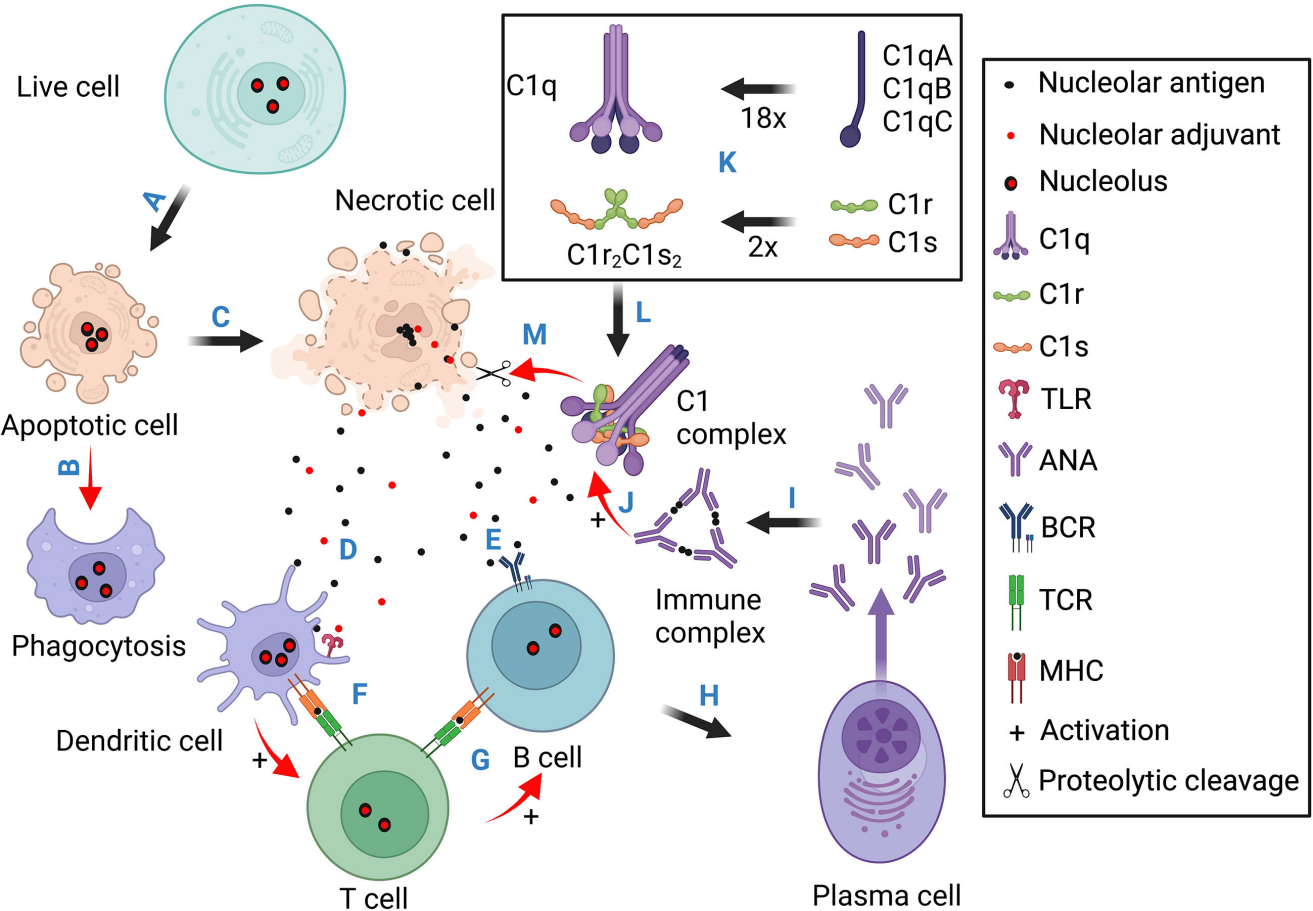
Schematic illustration for the pathogenic contributions of necrotic cell death, the nucleoli, and complement deficiency. This diagram contains four sections. Section 1 **(A, B)** stresses that normal apoptotic cells are cleared through phagocytosis without eliciting innate and adaptive immune responses. Section 2 **(C-I)** illustrates the scenario of necrotic cell death. The released nuclear antigens and alarmins activate T cells through dendritic cells which help antigen activation of B cell differentiation into ANA-producing plasma cells. **(J)** Immune complexes formed between ANA and nuclear. antigens activate Fc receptor- (not shown) and C1/complement-mediated inflammatory tissue injuries. Section 3 **(K)** illustrates C1q assembly from 18 polypeptide chains and its association with two C1r and two C1s to form the C1 complex. Section 4 **(L, M)** shows that after C1q binds to necrotic cell debris such as the nucleoli, it activates C1r and C1s into active proteases which then dismantle the exposed nuclear antigens and alarmins to reduce ANA induction. Basically, apoptotic cells are cleared in silence. Necrotic cells can expose both antigens and adjuvants to induce ANA production. When complement C1 is functionally intact, it can degrade nucleolar autoantigens and alarmins (e.g., NCL, FBRL, and GAR1) to reduce ANA induction and C1 deficiency, therefore, causing antinuclear autoimmunity.

*C1Q*, *C1R*, *C1S*, and *C4* deficiencies are rare, but they often cause monogenic SLE (164, 180–182). Among these strong SLE risk genes, C1q and the two serine proteases C1r and C1s exist as a pentameric C1 complex (C1qC1r_2_C1s_2_) (178, 179). When C1q binds to antibodies in immune complexes, it activates C1r and C1s, and the C1s protease then cleaves C4 to trigger the complement classical pathway (183, 184). In SLE, immune complexes are formed between ANA and nuclear antigens which trigger C1-mediated complement activation and inflammatory tissue injuries (**Figure 4**). The fact that C1 deficiency causes ANA production and SLE pathogenesis was for a long time considered a paradox until research found that C1q not only binds to immune complexes but also binds to apoptotic cells (185).

## 7 The dead cell-C1 axis in SLE pathogenesis

The formation of L.E. cells in SLE patients reflects excessive necrotic cell death in the patients, the accumulation of naked nuclei, and nuclear opsonization by ANA for phagocytosis (1, 186). The surge of blood DNA antigen during SLE disease flare also suggests necrotic cell accumulation (53). This status could result from excessive cell death or impaired phagocytic clearance of dying cells (187, 188). For example, necrotic cells release nucleosomes which are rendered autoimmunogenic by the alarmin HMGB1 (125). In mice, injection of UV-induced syngeneic apoptotic cells can cause ANA production (189). This could be partly explained by the ready release of autoantigens and alarmins by UV-induced dead cells, e.g., NCL, NPM1, HMGB1, and FBRL (131).

In 1997, C1q was reported to bind to apoptotic cells via the blebs (185). Subsequent studies focused on the hypothesis that C1q opsonizes apoptotic cells to enhance phagocytosis and regulate phagocyte responses (190, 191). Apoptotic cell disposal is mediated through multiple phagocytic pathways and that mediated by C1q is not dominant (192). On the other hand, C1q exists as a pentameric C1qC1r_2_C1s_2_ complex (178, 179), and how C1r/C1s deficiency also leads to monogenic SLE, like C1q deficiency, is not explained by the phagocytosis hypothesis (164, 181, 182). Recent studies suggest that C1r/C1s degrade nuclear autoantigens and alarmin proteins that are exposed by dead cells and bound by C1q.

On necrotic cells, C1q binding is not limited to the surface as it also binds intensely to the nucleoli (**Table 1**) (21, 130, 193). This activates C1r/C1s into active proteases which cleave numerous nucleolar proteins (59). In the complement system, C1s only cleaves three substrate proteins, but with a peptide library, C1s was found to cleave non-complement peptides that predicted many intracellular protein substrates such as HMGB1 (194, 195). HMGB1 can be released by necrotic cells or secreted by live cells, and it is indeed cleaved by C1s (195). The nucleolar autoantigens NCL and NPM1 and additional other proteins are also cleaved by C1 proteases (59, 193). This makes the C1 complex an extracellular surveillance mechanism over dead cell accumulation, and it functions through phagocytosis and proteolytic dismantling of autoantigens and alarmins to avoid nuclear autoimmunity (59). This helps explain why C1q, C1r, or C1s deficiency often causes monogenic SLE (181, 196).

## 8 Nucleolar autoimmunogenicity

The strong nucleolar autoantigenicity is characterized by the numerous autoantigens in this nuclear region, and the nucleolus is often the sole ANA-targeted region (51). With isolated nucleolar, nucleoplasmic, and cytoplasmic fractions, nucleolar proteins were found most frequently targeted by SLE patient ANA (59). Besides SLE, hepatocellular carcinoma patients also develop ANA that persistently target nucleolar proteins (148). This is not surprising for the large number of autoantigens in the nucleoli (**Table 1**) (21, 130). This is further explained by the prevalent (5-20%) nucleus-reactive naïve B cells in healthy individuals that express prominent nucleolus-reactive antigen receptors (120–122). When necrotic cells accumulate, the nucleolar antigens and alarmins could activate these B cells into ANA-producing B cells (122). This has been reported for the major autoantigens in the speckled region, i.e., U1-snRNPs, in which the U1-snRNAs were sufficient adjuvants to confer U1-snRNPs autoimmunogenecity (127).

In the nucleolar DFC region, the C/D box U3-snoRNP component FBRL has dual autoantigen and alarmin activities. In the H/ACA box U3-snoRNPs, the GAR1 component has adjuvant activity albeit autoantigen has not been reported in these complexes. In the nucleolar GC region, NCL also has dual autoantigenic and adjuvant activities (131). It would be interesting to test whether NCL and FBRL induce their self-reactive antibodies and whether these nucleolar alarmins are sufficient to confer autoimmunogenicity to the numerous other nucleolar and nucleoplasmic autoantigens.

In this context, studies on the clone 564 mouse autoantibody suggested an immunological pathway for autoimmunological epitope spreading (197). This antibody is cationic and polyreactive with single-strand DNA/RNA, nucleosomes, La/SSB, etc., and its IIF image showed intense nucleolar and cytoplasmic staining (197–199). Transgenic 564 expressions in C57BL/6 mice (564Igi) produced antibodies that stained the nucleolus (198, 199). In these mice, the transgenic B cells initiate spontaneous germinal centers in which other autoreactive B cells also proliferate to produce ANA of broader specificity (200). Whether NCL- and FBRL-reactive B cells similarly initiate autoreactive germinal centers need to be investigated.

## 9 Concluding remarks

The collective and individual significance of ANA has been testified by their increasing weightage in SLE diagnosis (50). However, answers remain fragmental with regard to what cause these autoantibodies, e.g., tolerance breakdown, dead cell accumulation, infection, etc. The prevalence of self-reactive naïve B cells in healthy individuals places particular importance on peripheral tolerance (121). The growing number of alarmins in the most autoantigenic nuclear regions, i.e., the chromatin network, the speckled regions, and nucleoli (**Table 2**), suggests their intrinsic capacity to overwhelm peripheral tolerance after necrotic exposure, and cause ANA production (123–128, 131). Necrotic cells are known to accumulate in SLE patients and release nuclear materials (1, 53). In this context, the four earliest components of the complement classical pathway, i.e., C1q, C1r, C1s, and C4, may be considered as an essential albeit insufficient tolerance mechanism against dead cell-induced autoimmunity (**Figure 3**) (181). These are rare genetic deficiencies that are not captured in most population studies, and the scarcity of these patients can be explained by the severity and early onset of the disease (166, 172). Nonetheless, these genetic deficiencies have offered a unique pathway of investigation into the causes of ANA and SLE pathogenesis.

**Table 2:** Possible nuclear triggers of self-reactive immunity.

Nuclear regions	Stimuli of the immune system		
	Adaptive immunity	Innate immunity	
		Adjuvants	Receptors
Chromatin network	dsDNA, nucleosome, and histones	HMGB1	TLR2, TLR4, TLR5, TLR9, and SAGE
mRNA synthesis	hnRNP, U1-snRNP, Sm, Pol III, SS-A/Ro, and SS-B/La	U1-snRNA	TLR7
rRNA synthesis	PM/Scl-75, PM/Scl-100, Th/To, NPM-1, NCL, No55/SC65, FBRL, Pol I, and UBF	NCL, FBRL, and GAR1	TLR2 and TLR4

The discovery of C1q binding to apoptotic cells formed the cornerstone of an immunological axis in understanding ANA induction and SLE pathogenesis. An initial hypothesis was that C1q opsonizes apoptotic cells for effective clearance to avoid immune exposure (**Figure 4**) (185, 190, 201). A more recent hypothesis is that C1q targets C1 proteases to dead cells to dismantle autoantigens and alarmins and therefore diminish their immunogenicity and avoid immune responses that lead to ANA production and immune complex-mediated tissue injuries (**Figure 4**) (178, 179). The observed C1q targeting to the highly autoantigenic nucleoli in necrotic cells (193) and C1s cleavage of nucleolar proteins (59, 131), i.e., autoantigens and alarmins (21, 59, 130), are in line with this hypothesis. Besides nucleolar proteins, the C1 proteases may broadly degrade and inactivate nuclear autoantigens and alarmins like HMGB1 (195).

At present, there is insufficient data to harmonize this hypothesis with how C4 deficiency similarly causes ANA and SLE (181, 182, 202). Based on the complement system, when C1s is activated on dead cells, it is expected to cleave C4 so C4b deposits on dead cells, and C4a is released as a weak anaphylatoxin (183, 184). C4b can target dead cells to phagocytes, B cells, and follicular dendritic cells through the complement receptor CD21/CD35 (183, 184), which is relevant to antibody induction. Carroll and colleagues reported that C4-deficient mice had a defect in transitional autoreactive B cell deletion and tended to form autoreactive germinal centers (198). It is possible that C4b-linked dead cell antigens inhibit autoreactive germinal center reactions and prevent antibody class switch by the prevalent self-reactive naïve B cells (120, 122). It has not been tested whether C4b-linked dead cell antigens are also cleaved more effectively because C2 is only effectively cleaved by C1s when it is associated with C4b. Further study of how C4 is related to this C1-dead cell axis of ANA induction and SLE pathogenesis could reveal more definitive underlying mechanisms for improved diagnosis and therapeutic targeting.

## Author contributions

JL initiated the article and contributed to the framework and major details of the final version. SW contributed to the details in nucleolar alarmins. JJC contributed to the details on nucleolar structures. BHDT contributed to the details in **Figure 4**. SYKW contributed to the details on B cells. JZC and JMC helped in bioinformatics that generated **Figure 3**. KPL contributed to the clinical aspects of this manuscript. All authors contributed to the article and approved the submitted version.
